# Novel model to authenticate role-based medical users for blockchain-based IoMT devices

**DOI:** 10.1371/journal.pone.0304774

**Published:** 2024-07-10

**Authors:** Muhammad Shehzad Aslam, Ayesha Altaf, Faiza Iqbal, Natasha Nigar, Juan Castanedo Galán, Daniel Gavilanes Aray, Isabel de la Torre Díez, Imran Ashraf

**Affiliations:** 1 Department of Computer Science, University of Engineering & Technology, (UET), Lahore, Pakistan; 2 Universidad Europea del Atlántico, Santander, Spain; 3 Universidade Internacional do Cuanza, Cuito, Bié, Angola; 4 Fundación Universitaria Internacional de Colombia, Bogotá, Colombia; 5 Universidad Internacional Iberoamericana Campeche, Campeche, México; 6 Universidad Internacional Iberoamericana Arecibo, Arecibo, Puerto Rico, United States of America; 7 Department of Signal Theory, Communications and Telematics Engineering, Unviersity of Valladolid, Valladolid, Spain; 8 Department of Information and Communication Engineering, Yeungnam University, Gyeongsan, South Korea; Jazan University, SAUDI ARABIA

## Abstract

The IoT (Internet of Things) has played a promising role in e-healthcare applications during the last decade. Medical sensors record a variety of data and transmit them over the IoT network to facilitate remote patient monitoring. When a patient visits a hospital he may need to connect or disconnect medical devices from the medical healthcare system frequently. Also, multiple entities (e.g., doctors, medical staff, etc.) need access to patient data and require distinct sets of patient data. As a result of the dynamic nature of medical devices, medical users require frequent access to data, which raises complex security concerns. Granting access to a whole set of data creates privacy issues. Also, each of these medical user need to grant access rights to a specific set of medical data, which is quite a tedious task. In order to provide role-based access to medical users, this study proposes a blockchain-based framework for authenticating multiple entities based on the trust domain to reduce the administrative burden. This study is further validated by simulation on the infura blockchain using solidity and Python. The results demonstrate that role-based authorization and multi-entities authentication have been implemented and the owner of medical data can control access rights at any time and grant medical users easy access to a set of data in a healthcare system. The system has minimal latency compared to existing blockchain systems that lack multi-entity authentication and role-based authorization.

## Introduction

The IoT (Internet of Things) extends the existing Internet into a network of geographically dispersed physical devices, enabling applications such as smart homes, remote surveillance, smart cities, and smart medical care, among others. It is projected that by 2025, there will be 41.6 billion IoT devices worldwide, and this number will increase exponentially in the years to come [1]. IoMT (Internet of Medical Things) is a promising IoT application that connects medical devices and applications to the healthcare system via the IoT network [2]. Reportedly, cardiac, kidney, posture, neurologically disabled, chronic, and aged patients are monitored by medical sensors using cellular, ZigBee, WiFi, and RFID (radio frequency identification) technologies to integrate and share data over the IoT network to reduce the effort required by medical users like doctors, medical staff, pharmacist, etc. The wearable sensors log data and provide real-time monitoring, allowing medical staff to monitor patients at home or while traveling for treatment.

Patients with chronic illnesses must be continuously monitored [3]; consequently, these patients rely heavily on wearable sensors to be remotely monitored. Medical sensors generate a large amount of data that can be used for research in various fields of medical science, and engineering to solve daily life problems [4] to benefit humanity [5], like study [6] uses machine learning algorithms to predict medical condition in real-time. Data generated by IoMT devices can be stored directly on cloud services such as Amazon Web Services or forwarded to fog for processing in a distributed environment, and then analyzed and shared [7].

Cloud-centric survey [8] indicates that IoMT healthcare systems rely on cloud computing for health records and medical services. Medical users, the patient, the hospital’s healthcare system, and the cloud are all components of cloud-based systems. Attached to the patient’s body are sensors that form a body area network to collect data regarding blood pressure, pulse rate, step counts, etc [9]. Joyia et al in [10] discuss the advantages, applications, and challenges of the IoT in the medical domain. Connecting wearable medical devices to clinics’ healthcare systems allows doctors to analyze patient data without human intervention and make decisions based on that analysis. Therefore, the patient does not need to carry his medical history and past reports to share with another physician. When a patient visits a healthcare center, he may need to see multiple medical specialists in the same healthcare center. As the patient must connect his monitoring devices to the hospital’s medical system to share data, and as the patient may change medical specialists, the process becomes more dynamic.

The intruders can attack the network through sensors, and intermediate layers or interfere with the communication messages while the patient is sharing his medical data or during normal operation of sensors. Viraj et al [11] demonstrate such an adversarial attack by sensor spoofing and by issuing incorrect commands to actuators. They interface the feedback control system on the cyber-physical system to gain access to the controller component to highlight the security issues. This issue becomes worse with the dynamic IoMT network. The dynamic connectivity of IoMT devices exposes data confidentiality, integrity, and privacy to security vulnerabilities [7, 12, 13]. As the intruder can exist anywhere hence, patient data must be protected to ensure privacy.

Due to built-in security and traceability features, blockchain become popular to solve the issues of data privacy and security. Data in the blockchain is stored in the distributed ledger and the record of transactions is maintained by multiple nodes in the blockchain network. The consensus process is adopted by nodes to agree on the state of data to commit transactions on the blockchain. Blockchain is being used now in various fields like medical, transportation, agriculture [14, 15] etc. As patient data is sensitive, the blockchain becomes an integral choice for medical healthcare systems. The literature proposes the use of blockchain-based authentication tools to save patient data from malicious users [16]. These systems use blockchain to work on network edge level [17] and the cloud level along with machine learning features [18] to solve the issues of privacy and security. Blockchain successfully provides data privacy and security to monitor COVID-19 patients [19].

The challenging problem of privacy is somehow solved by blockchain technology, but when medical data have varying levels of access, the problem of privacy and access becomes more complex. The medical staff may require a subset of the data generated by the sensor, whereas doctors or experts may need a different set of data. In addition, each medical user must be individually authenticated in order to gain access to the medical data. As patient medical information is private and could be misused or interfere with one’s personal life if compromised, it must be protected from all aspects. Therefore, it is necessary to define a more efficient authorization framework that allows medical users to access only a subset of data. The existing studies try to overcome the issue of privacy by proposing various authorization models to grant access to the full data of patients or divide the data over the medical users. Existing studies require the patient to authenticate the medical users individually, which increases the administrative burden. Individual authentication may work if patients want to see one medical user, but most of the time patients need to see multiple medical experts in the same healthcare center.

In this study, we introduce a new approach for role-based entity authentication to ensure the authorization of medical users with IoMT devices in order to protect patient data privacy in a dynamic environment. Medical users within a hospital’s MHS (medical health care system) are reliable entities. To reduce the administrative burden of authenticating each medical user, we developed a method that automatically authenticates medical users within the MHS based on trust models. The access rights of authenticated users are defined by the data owner. In conclusion, the following are major contributions of this study. First, the data generated by medical sensors will be stored on the blockchain to provide security for patient information. Second, a role-based access control is proposed. The proposed model authenticates medical users to grant them access to patient data based on their individual requirements. Third, multiple authentication entities are used to reduce the administrative burden of granting access to multiple medical users. We propose a method for medical users to authenticate themselves via their MHS in order to request patient data. Lastly, due to the dynamic nature of the data and the frequent visits of medical users, the data owner can grant or revoke access to any medical user or MHS at any time.

This study is divided into six sections. Section 2 contains a review of the literature. Section 3 presents the proposed methodology. Section 4 discusses the attack model and security analysis of the proposed framework. In section 5, results and discussion are presented. Section 6 concludes with a discussion of potential future directions.

## Literature review

With the expansion of IoT applications, security threats are also on the rise [20], and IoT systems must be protected against all potential risks and threats. As IoT devices can be remotely controlled and accessed by an adversary, the authentication concern in the IoT ecosystem is one of the primary security threats that must be addressed [21]. IoT architecture is complex and does not have a unified security protection mechanism hence, privacy issues occur during data transmission. IoT devices are limited in computational power, hence different techniques are required to protect the data in IoT networks. In cloud-based networks, protection techniques can reduce efficiency, introduce delays, and consume sensor-limited energy. The study [22] uses a machine learning technique at fog to reduce latency between the sensor and cloud, and study [23] proposes a data aggregation technique between IoT sensors and cloud to save sensor resources as well as provide security. To secure communication in IoT networks, DTLS (datagram transport layer security) is recommended over TLS (transport layer security) in constrained environments, but the study conducted in [24] shows that DTLS is vulnerable to DoS (denial of service) attacks and they proposed enhanced lightweight DTLS that overcomes the shortcoming of conventional DTLS by introducing a trusted third party. In a trusted network, nodes are free to establish trust relationships to exchange data. However, IoT devices may rely on insecure communication channels, so separate techniques for security and authentication have been proposed in the literature [13, 25].

In an IoT environment, remote user authentication is the validation of a legitimate user’s credentials by a remote node over an insecure communication channel [21, 26]. Once the user’s identity has been confirmed, he or she is granted permission to utilize the permitted resources. Authentication not only distinguishes between legitimate and illegitimate users but also ensures the transparency of IoT transactions [26]. The use of character passwords is the simplest method of user authentication [26]. However, this method has several drawbacks including being difficult to remember and easy to forget, guessable, and susceptible to dictionary attacks, among others. Biometric authentication methods have been proposed in the literature as an alternative to password-based authentication. Biometric authentication, such as the use of facial features, fingerprint scans, etc., offers numerous benefits. Biometric characteristics are difficult to forge, steal, or forget and are unique to each individual [27]. A smart card is another method presented in the research literature for overcoming password authentication problems [26]. However, smart cards are highly susceptible to theft and loss. Numerous studies advocate a combination of user authentication methods for enhanced security [28]. Two-factor authentication refers to any combination of two methods, such as the use of passwords and biometrics or the combination of passwords and smart cards. 2016 saw the compromise of two-factor authentication for numerous mobile banking applications [29]. To increase security, the two-factor approach is expanded to three-factor user authentication [28, 30].

Most of the data security techniques in IoT work on textual data. Sensors in IoT networks can generate images too, and the said techniques may not be applicable to images. Visual Cryptography (VC) technique can be used to protect images during transmission in an IoT network. Study [31] performs VC technique along with pre-trained on high-resolution remote sensing images to increase the performance.

The provision of a privacy and authentication scheme for cloud-based medical environments has been the subject of research [32]. Nevertheless, the scheme has been subjected to crypt analysis and found to be insecure and failing to ensure patient anonymity [33]. In response, a 1024-bit key-based smart service authentication framework was proposed. Earlier schemes relied on a central system to store patient data, which could be compromised if the central system’s security was compromised. The cloud computing concept is not recommended for IoMT healthcare systems for a variety of reasons, including increased latency, storage cost, and a single point of failure [34]. Due to its decentralized, transaction-based architecture, blockchain has gained popularity as a means of providing data security. As the removal or alteration of blockchain participants has no effect on the remaining parties, blockchain has gained popularity in medical applications [35]. Xing et al. [36] used blockchain features to address security and privacy concerns by providing anonymity, traceability, etc., by generating mutual keys based on time stamps and random primes. Arual [37] propose a blockchain-based model to store the keys in a blockchain smart contract and the data is encrypted using the keys to be stored on the cloud, further they developed a mechanism to share the patient data with doctors, however, data remains on the cloud. Sharma [38] uses blockchain to store medical documents of patients so that they do not carry physical documents and provides a mechanism to access the documents by another user. There have been efforts to develop more robust authentication and privacy schemes. Akkaoui [34] combined PUF (physical unclonable function) and blockchain for more secure device authentication. The study [12] proposed a three-layered (produce, consumer, and computing) security and privacy scheme based on blockchain and smart contract-based services. Due to low throughput, high latency, and resource consumption, blockchain is discovered more slowly. Akkaoui et al. [39] proposed a four-layered framework that combines edge computing and blockchain characteristics. For faster processing, blockchain transactions are computed at the network’s edge. Similarly, [40] utilized a blockchain-based public key table to move processing to the fog.

Once a user has been authenticated, he must have access to patient data. In a shared environment, access control is the technology that monitors user rights and prevents the flow of information to unauthorized parties. Role-based, attribute-based, and capability-based access control models are well-known in the literature, and nearly all access control models are based on these models. The study [41, 42] extends the role-based access control model to secure data on the cloud, while [43] utilizes the blockchain’s capabilities to provide attribute-based access control. Zhang et al. [44] proposed the implementation of smart contract-based access control on the Ethereum platform. Similarly, [45] presents smart contract-based access control for IoT entities without a central authority. The transmission of data from one node to another with authorized access control and predefined attributes is facilitated by blockchain in this context.

Recently, Covid-19 has had such a global impact that the WHO has classified it as a pandemic. The pandemic sparked an entirely new discussion about how IoMT can assist medical users in their day-to-day activities [5, 19, 46]. The use of wearable sensors to store data in the cloud for remote monitoring of COVID-19 patients raises privacy and security concerns. The research is summarized in Table 1. To address the aforementioned difficulties, we propose a role-based authorization model to authenticate medical users within a medical healthcare system.

**Table 1:** Comparative analysis of published authentication schemes.

Reference	Scope of study	Approach	Summary	S	R	M	D
[26]	User authentication in IoT	SLR	Summarize the existing studies	Yes	No	No	No
[28]	User authentication in IoT	Biometric	Relay on biometric features to authenticate users	Yes	No	No	No
[27]	User authentication in IoT	Three-factor authentication	A more secure scheme than biometric	Yes	No	No	No
[30]	Remote user authentication in IoT	Three-factor authentication	A more secure scheme than biometric for remote users	Yes	No	No	No
[32]	Security in healthcare cloud	Crypto Algorithm	Algorithmic approach for securing data	Yes	No	No	No
[33]	Security in healthcare cloud	Crypto Algorithm	New algorithmic approach to overcome flaws in [32]	Yes	No	No	No
[36]	User authentication in Healthcare	Blockchain	BAN logic and NS tool for simulation	Yes	No	No	No
[45]	Access control of user	Blockchain	Define access algorithms and Implement using Ethereum in solidity language. No experiments or results	Yes	Yes	No	No
[39]	IoMT	Blockchain and Edge computing	Implemented using Ethereum and solidity. Measured transactions per unit time, scalability, latency with other schemes	Yes	No	No	No
[7]	Edge based IoMT	Blockchain	Processing at edge	Yes	No	No	No
[13]	IoMT	Blockchain and PUF	Physical layer security over centralized server	Yes	No	No	No
[34]	IoMT	Blockchain and PUF	Ethereum-based blockchain using the Geth client. Synchronization, encryption overhead, and frequency of scalability of the ledger are measured	Yes	No	No	No
[40]	IoMT	Blockchain based public key table on fog computing	Used Ali cloud for simulation. Compared computation cost in terms of comparison and time	Yes	No	No	No
[38]	Security of Healthcare records	Interface between Blockchain and users	Provide distributed application between the blockchain and the end users to access medical documents	Yes	No	No	No
This study	IoMT	Blockchain based trust certificates and smart contracts	Role-based access rights and trust certificates in smart contract	Yes	Yes	Yes	Yes

S = Security, R = Role-based authorization, M = Multi-entities authentication, D = Dynamic rights

## Methodology

**Fig 1:**
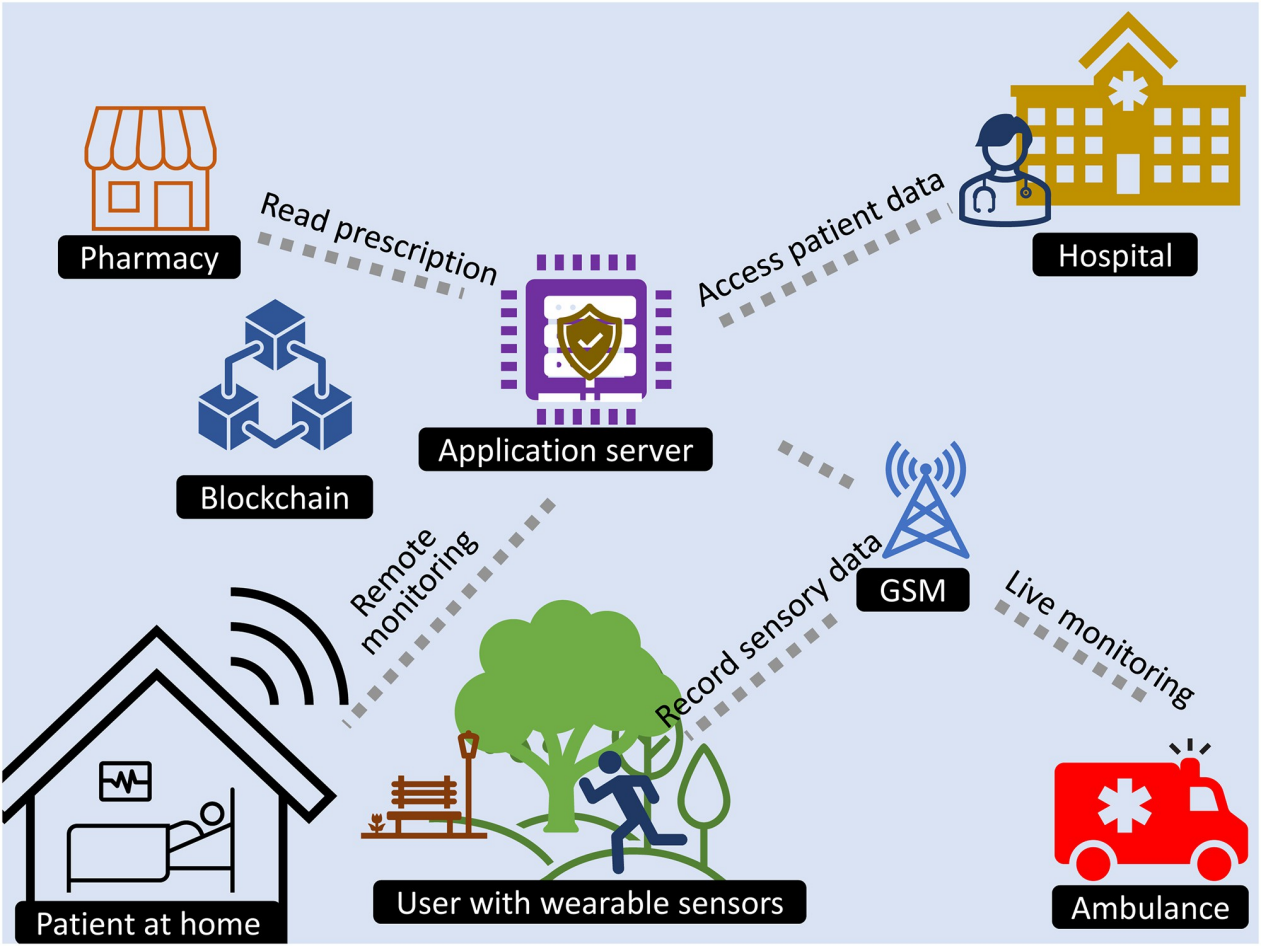
Overview of the IoT medical echo system based on blockchain.

**Fig 2:**
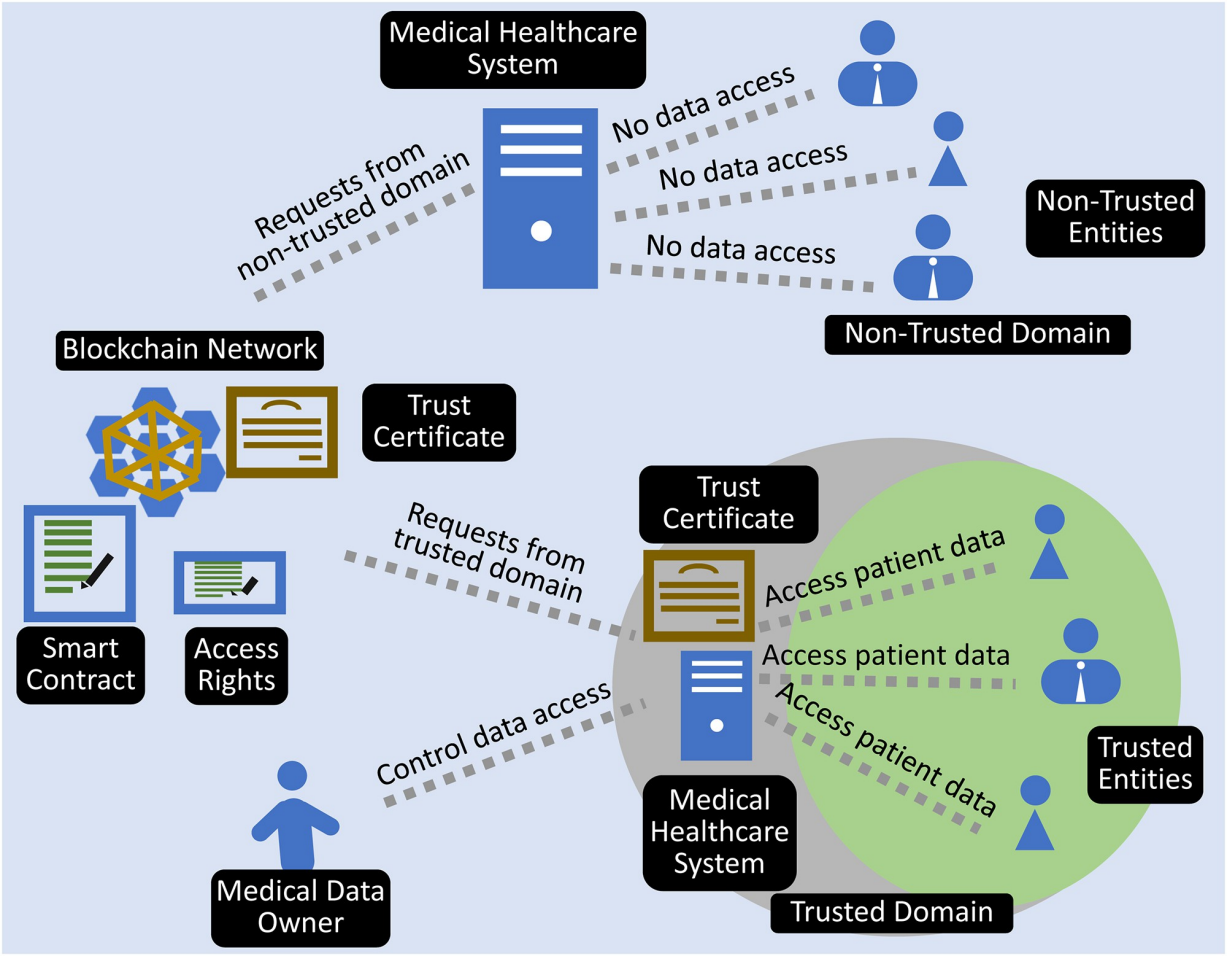
Proposed model for multi-entity authentication and role-based authorization.

Smart devices and wearable sensors are connected over an IoT network to store the data in the cloud. In the blockchain environment, the data is stored on the distributed ledger as shown in Fig 1. This section describes the proposed authentication scheme for medical users to access patient data in a blockchain-supported environment. The model includes four entities as presented in Fig 2.

- Medical data owner (patient, ambulance, public),
- Medical users (doctor, nurse, lab technician, pharmacist, etc.),
- The Blockchain (smart contracts, trust certificates, access rights, distributed ledger),
- Medical healthcare system of hospital

### Medical data owner

The MDO (medical data owner) is the patient or ordinary user *p*_*i*_ ∈ *P* who employs medical sensors to generate data *d*_*j*_. Medical sensors are attached to a person’s body to create a body area network. Each sensor *s* generates the data *d*_*j*,*s*_ to be stored on the blockchain, and sensors continuously monitor a person’s physical condition. The owner of medical data deploys a smart contract on the blockchain, at which point he becomes the owner of the data. The MetaMask account address serves as an identifier for data owners and medical system users.

$$\begin{array}{c} P=\{p_{1},p_{2},p_{3},\ldots ,p_{k}\} \end{array}$$

$$\begin{array}{c} D=\{d_{1},d_{2},d_{3},\ldots ,d_{L}\}L\leq k \end{array}$$

$$\begin{array}{c} d_{j}=\left\{d_{\left(j,1\right)},d_{(j,2}\right),d_{\left(j,3\right)},\ldots ,d_{\left(j,s\right)}\} \end{array}$$

$$\begin{array}{c} \text{Let}\;d_{j}\in D,\text{then}\;\exists p_{i}\in P|d_{j}\in p_{i} \end{array}$$

When a medical user needs access to the data, he must submit a request to the data’s owner. The owner of medical data assigns access permissions to medical users for their subset of data. When the data owner visits the hospital or clinic, it grants access to the MHS. The hospital’s medical users can rely on the trust certificate to access patient information.

### Medical users

Medical users are consumers of medical data, and they analyze the information to make decisions. The medical data owner must authenticate medical users before granting access to a patient’s medical data. The process is arduous because the patient may have to visit multiple medical providers during the examination. Each medical user requires a unique subset of data, and access must be granted beforehand. The framework enables medical users to obtain patient authentication through their hospital’s MHS. Consequently, each medical user within the trusted context has access to the patient’s data.

### Blockchain

Patient information is stored on distributed blockchain ledgers which are protected by smart contracts that regulate data access and privacy. The owner of the medical data dynamically generates role-based access rights for the medical users. The owner of medical data deploys these smart contracts to acquire ownership of the data. Only the data owner has the ability to assign roles and rights to medical users. At any time, the data owner may revoke access for any medical user. To ensure confidentiality, medical users are authenticated in the context of these rights.

When the owner of medical data needs to grant access to a medical user, he creates a binding between the medical user and the created set of roles/users. The medical user initiates the binding procedure by submitting a request to the data owner. The data owner grants access and assigns a role to the medical user, creating a binding within the smart contract. These bindings are keys to implementing partial and role-based access to the patient data. The binding can be defined as if *p*_*i*_ grants *p*_*u*_ access to his data *d*_(*j*, *s*)_ for a period of time *t*, then a binding *b* has occurred.

$$\begin{array}{c} b=d_{\left(j,s\right)}\overset{p_{i}}{\underset{t}{\to }}p_{u} \end{array}$$

This binding in smart contracts ensures that access to the subset of data is granted. The owner of medical data is not required to individually grant access to medical data users.

### Medical healthcare system

The hospital owns a medical healthcare system that allows medical users to connect and carry out daily activities. As MHS keeps track of medical users and authenticates them with MHS, each medical user is a trusted entity for MHS, but these users are not trusted by the patient unless the medical data owner issues MHS a trust certificate. When the owner of medical data visits the hospital, he authenticates the hospital’s MHS at the information desk. After successful authentication, the MHS receives a trust certificate that medical users within the MHS can use to become trusted entities. After the issuance of a trust certificate, MHS becomes a trusted domain, and all entities within that domain become trusted entities for the owner of medical data. According to the access rights, trusted entities can have access to the medical data of the data owner.

### Authentication process

When a patient visits the hospital, he must visit the information desk to start the authentication process. The staff member at the information desk requests the patient to grant MHS access. The request appears on the patient’s mobile device as the information system transmits the roles that exist in their medical system to the patient, who then sees the corresponding request. The patient then uses biometric authentication to gain entry to the MHS. Accordingly, binding between the medical healthcare system, trusted parties, and user data occurs based on access rights. After successful binding, the system issues a trust certificate to the MHS for a specified period of time and grants the authority to authenticate trusted parties within the MHS. After the issuance of a trust certificate, the MHS becomes a trusted domain, and each medical user within that domain becomes a trusted entity. As demonstrated in Fig 2, now every trusted entity can use the issued trust certificate to authenticate with medical devices without the involvement of the medical data owner.

**Fig 3:**
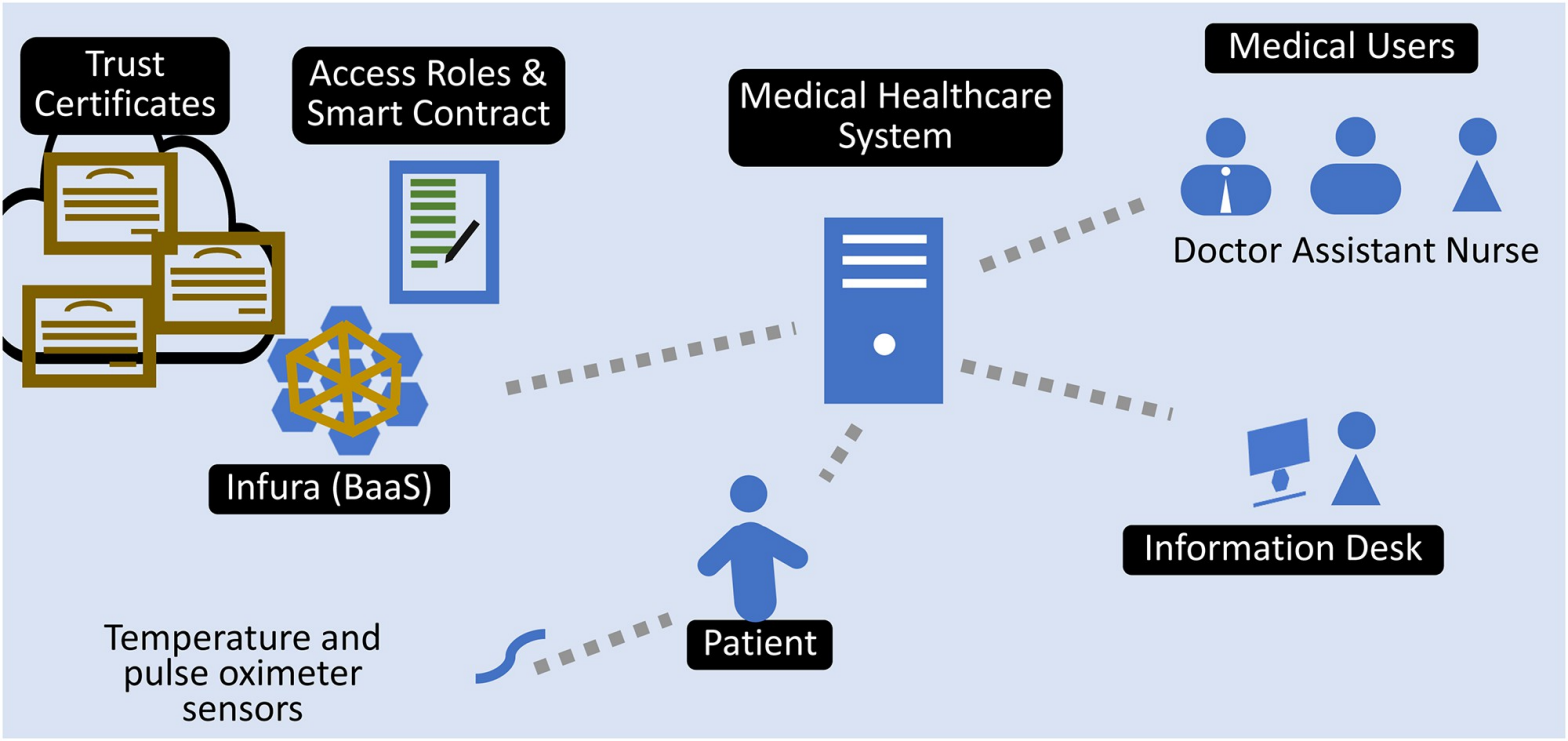
Access controls for medical users through the medical healthcare system.

Smart contracts ensure that confidentiality and access are provided for the subset of medical data required by the user. After the patient grants access, the medical system retrieves the patient’s basic information, which then appears automatically in the hospital’s medical system. When the patient has completed the checkup, he will revoke access to the medical system or the access rights will be revoked when the trust certificate expires. The system invalidates the trust certificate, de-authenticating all trusted parties within the medical system. The entire procedure is summarized in Fig 3.

## Attack model

**Fig 4:**
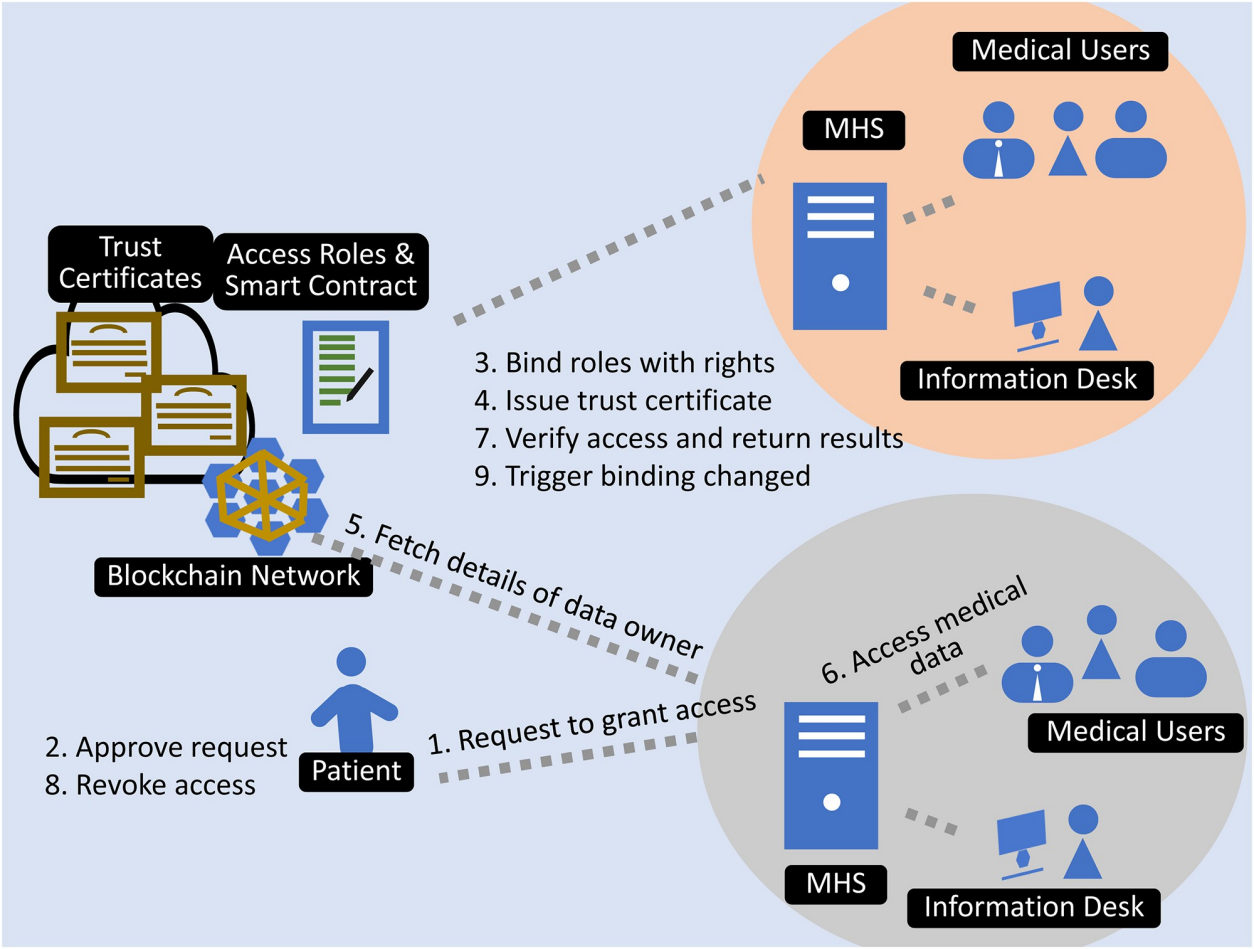
Attack model.

The medical healthcare system of the hospital acts as the middle party to request access to data from the medical data owner. The data owner grants access to the MHS based on the trust and assumes that every entity inside the MHS will remain a trusted entity. The problem is that the adversary can join the MHS to act as a medical user to process malicious requests through the MHS. The goal of the adversary is to gain access to the medical data or flooding the MHS with so many requests to launch a DoS attack. When the adversary requests to access data through MHS, it is considered a request from a legitimate medical user. The attack model is shown in Fig 4.

### Security model

In this section, we provide a security analysis of the proposed framework to show resilience against the adversary. Theorem 1 and Theorem 2 are related to security and confidentiality. Theorem 3 is related to the adversary model and Theorem 4 states how the framework prevents DoS attack on MHS.

**Theorem 1**. *When a medical user interacts with a data owner or vice versa, they communicate with legitimate users*.

*Proof*. The framework is protected from man-in-the-middle and reply attacks, as every request to read medical data is digitally signed on the blockchain. The signing process at MHS also involves the trust certificate IDs and timestamps to ensure that the legitimate medical user from the legitimate MHS is being communicated and that data is not tampered with.

**Theorem 2**. *Communication between medical users and data owners is confidential. The intruder can not access the medical data*.

*Proof*. The Ethereum Metamask address is backed with a-symmetric keys. The results of a request are encrypted using the public address of requesting medical users. This technique restricts any illegal access to the data of patients.

**Theorem 3**. *An adversary cannot rely on the trust certificate of MHS to gain access to the medical data*.

*Proof*. During the binding process, the medical users in MHS appear to the data owner for approval. These users are legitimate as given in Theorem 1. The binding *b* takes place between the data component *d*_(*j*, *s*)_ and medical user *p*_*u*_. To match the binding, the request must be signed by the *p*_*u*_. If the adversary becomes a part of MHS to sign its request through an issued certificate, the relevant binding *b* will be missing in the blockchain, and the request will be denied. This technique restricts any illegal access to patient data.

**Theorem 4** *The intruder cannot overwhelm MHS resources with flooding*.

*Proof*. The requests to access data in the MHS are processed in the queue that grows when the number of requests increases per unit of time. Requests from legitimate medical users are entertained in the request queue as well and a counter-check based on timestamps is maintained at MHS to control the resources. When the request arrival rate from a certain medical user exceeds the processing time, then MHS gives feedback to the medical user in the form of wait signals. The timestamp in the wait signal indicates that the next request form that the user will be entertained after the said time, and any subsequent request will be dropped. This technique puts a limit on the number of requests from a certain user to avoid any overwhelming resources. Hence adversaries become unable to block legitimate users from accessing data.

## Experiments and results

We used Remix to write smart contracts in the solidity programming language and deployed them on Infura in order to conduct the experiments. The experiments simulate the interactions among the users to demonstrate the operations in terms of security. Infura blockchain uses a proof-of-stake (PoS) consensus algorithm which is energy efficient.

### Experimental setup

**Fig 5:**
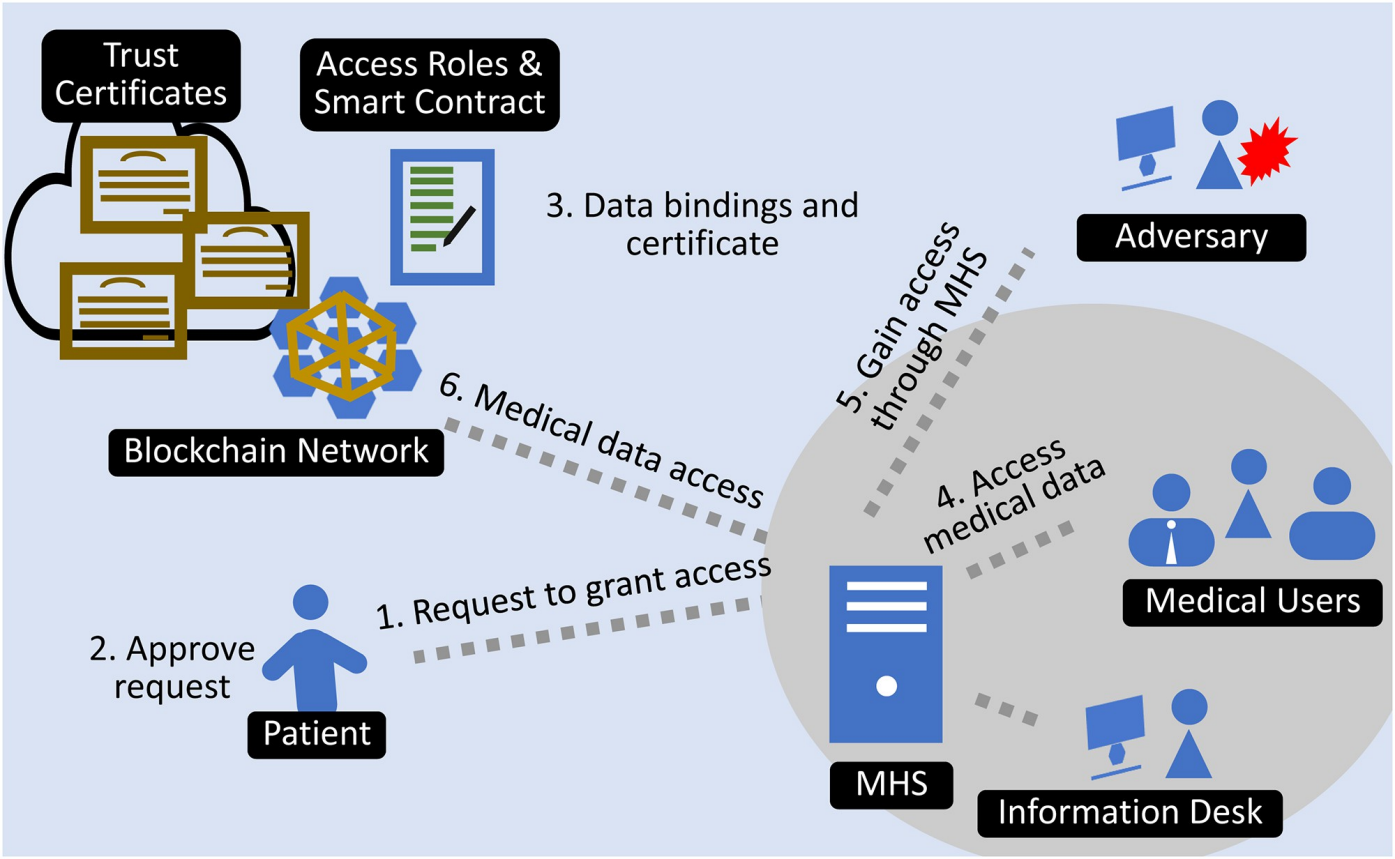
Experimental setup.

In the smart contract, the trust certificate and roles have been implemented. Medical users and data owners register on MetaMask to conduct blockchain transactions. Temperature and pulse rate sensors were attached to the patient’s body to generate the data. Fig 5 shows the experimental setup used for experiments.

A Python application logged the data generated by these sensors. The patient’s MetaMask account was used to sign the generated data for storage on the blockchain. For the experiments, the system assumes three types of medical users doctor, nurse, and assistant, and defines access rights for each medical user. Core-i7 systems with 12GB RAM (random access memory) were utilized for MHS, while core-i3 systems with 4GB RAM were used to deploy medical applications for medical users. Using a 100Mbps LAN (local area network), medical users and MHS are linked to one another.

**Fig 6:**
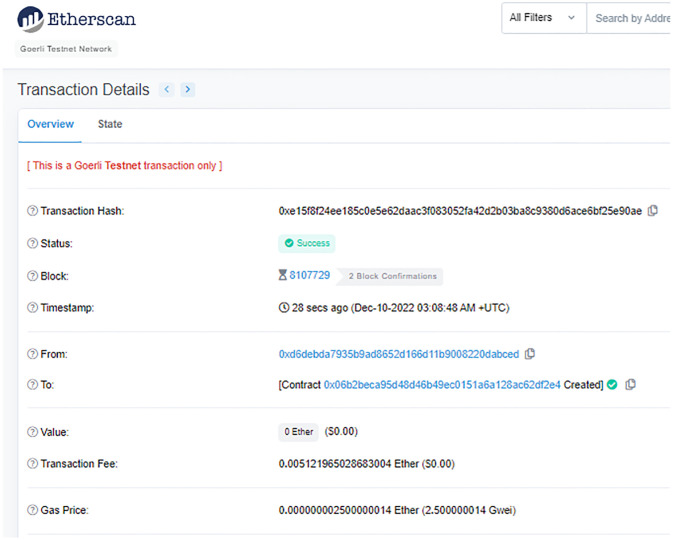
Transaction of Smart contract deployed by patient.

The doctor requires the patient’s medical history as well as the patient’s current condition as monitored by sensors; the nurse requires the patient’s current medical condition; and the assistant requires only the patient’s current temperature. In order to conduct the experiments, ten individuals of each type are registered in MHS. The smart contract is deployed by the patient, as shown in Fig 6, using his MetaMask account, and medical user roles were created in the smart contract as given in Table 2. After waiting a half-hour for the sensors to generate data, the following experiments were conducted.

**Table 2:** Medical user access rights to the experimental setup.

Medical user	Access temperature	Access pulse rate	Temperature history	Pulse rate history
Doctor	Yes	Yes	Yes	Yes
Nurse	Yes	Yes	No	No
Assistant	Yes	No	No	No

### Experiment 1: Authenticating MHS

This experiment simulated the authentication of multiple entities within the MHS using a certificate of trust. At present, no trust certificate is issued to the MHS, and the status of the MHS is that of an untrusted domain, with each entity within the MHS being an untrusted party for the patient. Any attempt to access patient data will be denied by the smart contract, as no trust certificate for the MHS that would allow medical users access to the data could be located.

**Fig 7:**
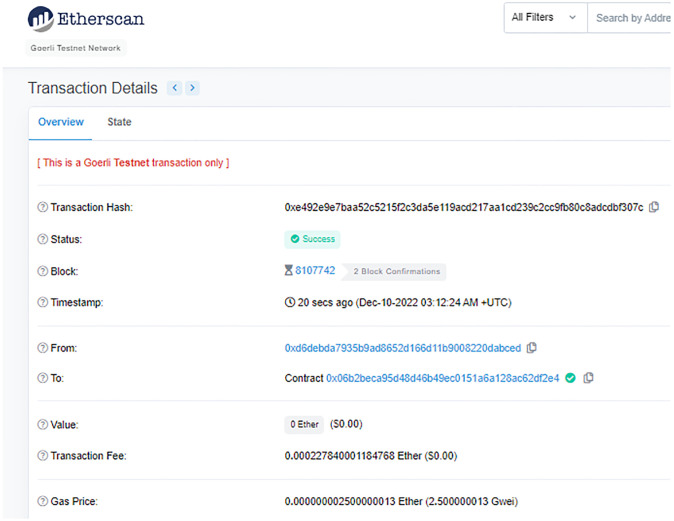
Transaction involving medical users and a trust certificate.

The receptionist then initiated a request for the patient to grant access to MHS medical users. The request included a list of medical users and the required rights to the data. As soon as the patient approved the request, medical users in MHS were bound to the patient data based on their access rights. This binding in the form of a trust certificate is recorded on the distributed ledger. The system then signed the trust certificate and issued it to the MHS so that it could become a trusted domain for 30 minutes. The MHS retrieved patient information, and the patient appeared in the MHS. The successful issuance of the trust certificate verifies that MHS has become the trusted domain and the entities that reside within the MHS have become the trusted entities. The patient does not need to grant access rights individually to medical users in the MHS and these medical users now can access the patient data according to the access rights. Any subsequent request for patient data can be validated by MHS locally through the issued trust certificate, and the blockchain will process the requests according to the bindings. Experiment 2 was conducted to ensure that all trusted entities within the trusted domain have access to the medical data based on the dynamic rights assigned by the patient. Fig 7 depicts the transaction of trust certificate binding.

### Experiment 2: Validity of trust certificate

To ensure the validity of the trust certificate, we repeated Experiment 1 and then attempted to access patient data from all authorized medical users. Since the trust certificate was valid, the requests were handled by the smart contract. We waited for a half-hour for the trust certificate to expire. Then, we attempted to access patient data from a previously trusted entity (doctor), but he was unable to access any patient data. The smart contract looked for the reference to the trust certificate but found that it was invalid. As a result, the transaction was rolled back with an error message indicating that the entity must grant access rights. This experiment verifies that multi-entity authentication is achieved with a trust certificate and that data confidentiality is maintained when the trust certificate expires. The inclusion of time constraints on the validity of the certificate makes sure that access is granted within time constraints which can be adjusted by the patient. The patient does not need to revoke access for the individual medical user, the validity of the trust certificate successfully reduces the administrative burden on the patient. The validity of the certificate can also be managed by the patient as discussed in Experiment 5.

### Experiment 3: Verification of role-based access rights

**Fig 8:**
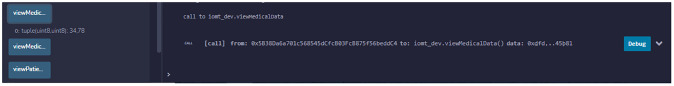
Patient temperature accessed by a doctor.

After issuing a certificate to the MHS, we conducted experiments to ensure that medical users could access data within the scope of their granted permissions. The doctor accessed the patient’s medical history; Fig 8 demonstrates that the transaction was successful, and the patient’s medical history is displayed on the doctor’s screen.

**Fig 9:**
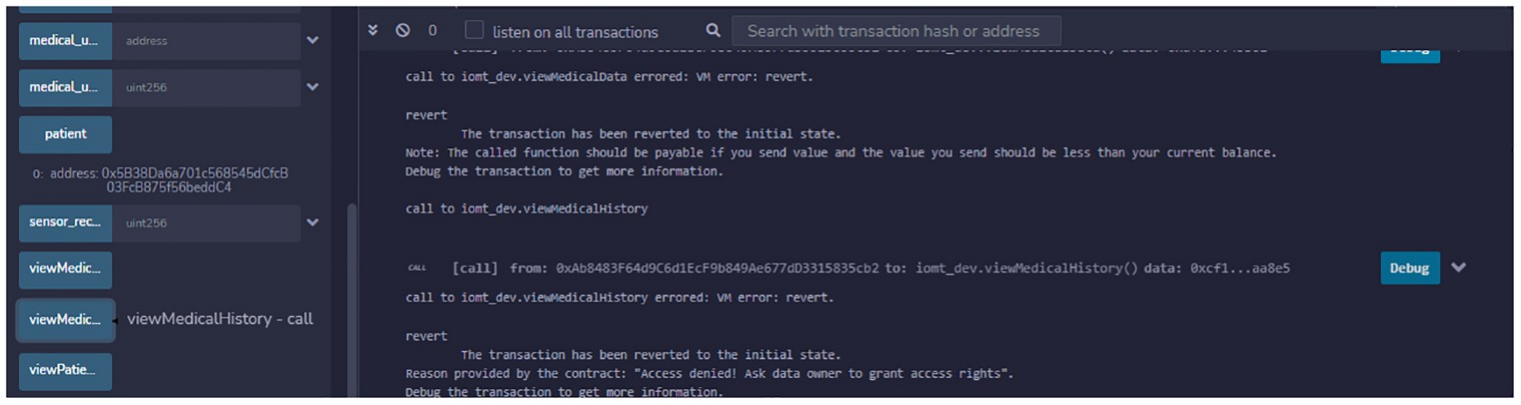
Patient medical history accessed by nurse.

We conducted a second experiment to confirm that the system restricts user access to data when they exceed their permissions. In this instance, the nurse attempted to access the patient’s medical history, but the smart contract denied the request because it could not find any access right in the trust certificate binding the nurse against accessing the patient’s medical history. Consequently, the transaction is rolled back with an error message stating that you do not have permission to access the data, as shown in Fig 9. This experiment verifies the achievement of role-based authorization is successful. The system does not expose full data to any trusted entity and access to the data is granted on specific data parts only. The patient has sole authority to grant access to his data. The role-based access right ensures that privacy and data remain confidential.

### Experiment 4: Verification of revoking rights of single medical user

We conducted an experiment to demonstrate that a patient may at any time revoke the rights of an individual medical user. For this purpose, we first repeated experiment 1, and then the patient revoked the assistant’s access rights. Then, the assistant attempted to determine the patient’s temperature. The transaction failed with an error message stating that you lack access permissions to the data. This experiment confirms that patients can dynamically manage their rights and that patient data confidentiality is ensured. As the patient can revoke access rights at any time, hence he has exclusive ownership rights on their data.

### Experiment 5: Verification of invalidating trust certificate

**Fig 10:**
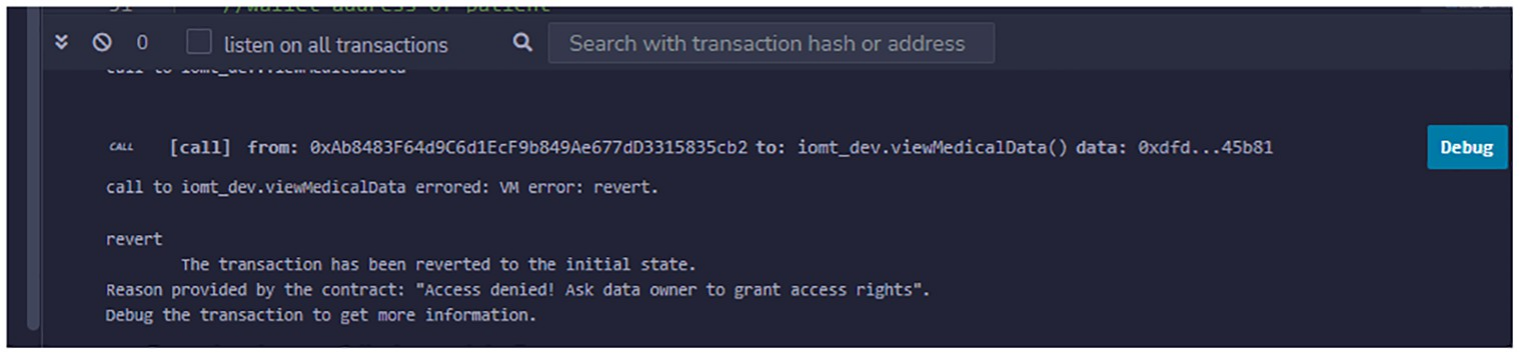
Patient data access after revoking access rights.

As a patient can invalidate the entire trust certificate to revoke access privileges for all trusted entities within the MHS, we conducted an experiment to confirm this phenomenon. In this scenario, we repeated experiment 1 before the patient invalidated the MHS’s trust certificate. The doctor then attempted to access patient data. The transaction is rolled back with an error message stating that you do not have permission to access the data in question, as given in Fig 10. The other users’ requests are also denied because the trust certificate has become invalid and the MHS domain is no longer trusted. This experiment verifies that when a trust certificate becomes invalid, multiple entities are automatically de-authenticated. There is no longer any connection between the data and medical users.

The aforementioned experiments and transaction status demonstrate that the model is capable of authenticating multiple entities based on the issued trust certificate and the patient does not need to grant access rights individually. Through data binding, each entity could access patient data within the scope of their granted access rights, and full data is never exposed to protect data privacy. The patient could revoke access rights for a single medical user or the entire MHS. The results confirm the model’s functionality in terms of authentication and authorization while protecting patient data privacy. The patient has exclusive rights to his data which he can grant or revoke dynamically or the rights are revoked automatically when the validity of the trust certificate expires.

### Experiment 6: Latency

The burden of processing each request against trust certificates and data binding can hinder response rates. This section describes the experiments conducted to determine the effects of bindings for role-based permissions and trust certificates.

#### Effect of access rights and multi-entities authentication

**Fig 11:**
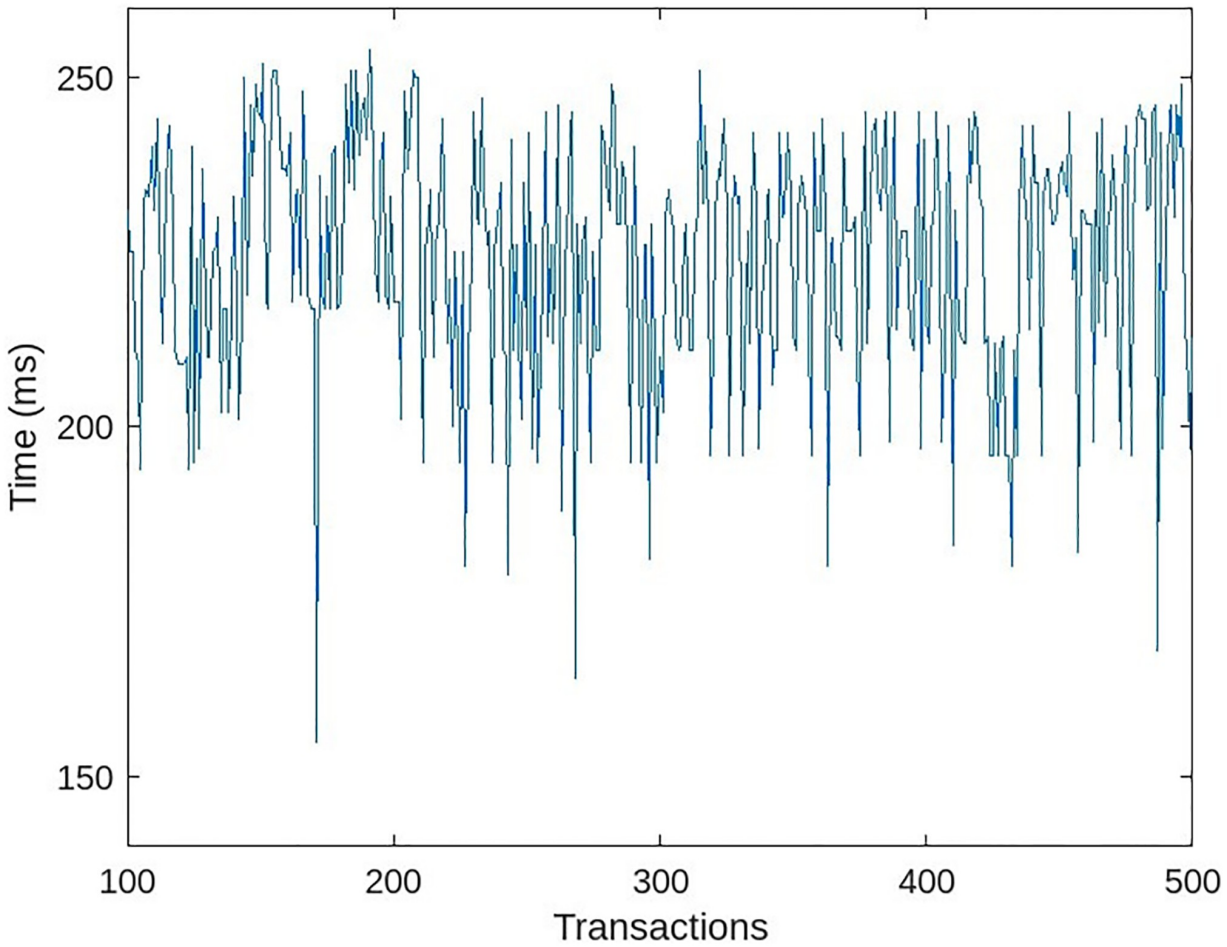
Accessing patient data without role-based access module.

To determine the impact of role-based access rights, we conducted two experiments with comparable outcomes. The initial experiment involved removing the access rights module entirely. Now, each medical user has full access to patient information. All thirty medical users in the system simultaneously accessed patient medical data from the blockchain. We increased the number of access transactions per unit of time over time. The latency of data access from the blockchain with the role-based access module disabled is depicted in Fig 11.

**Fig 12:**
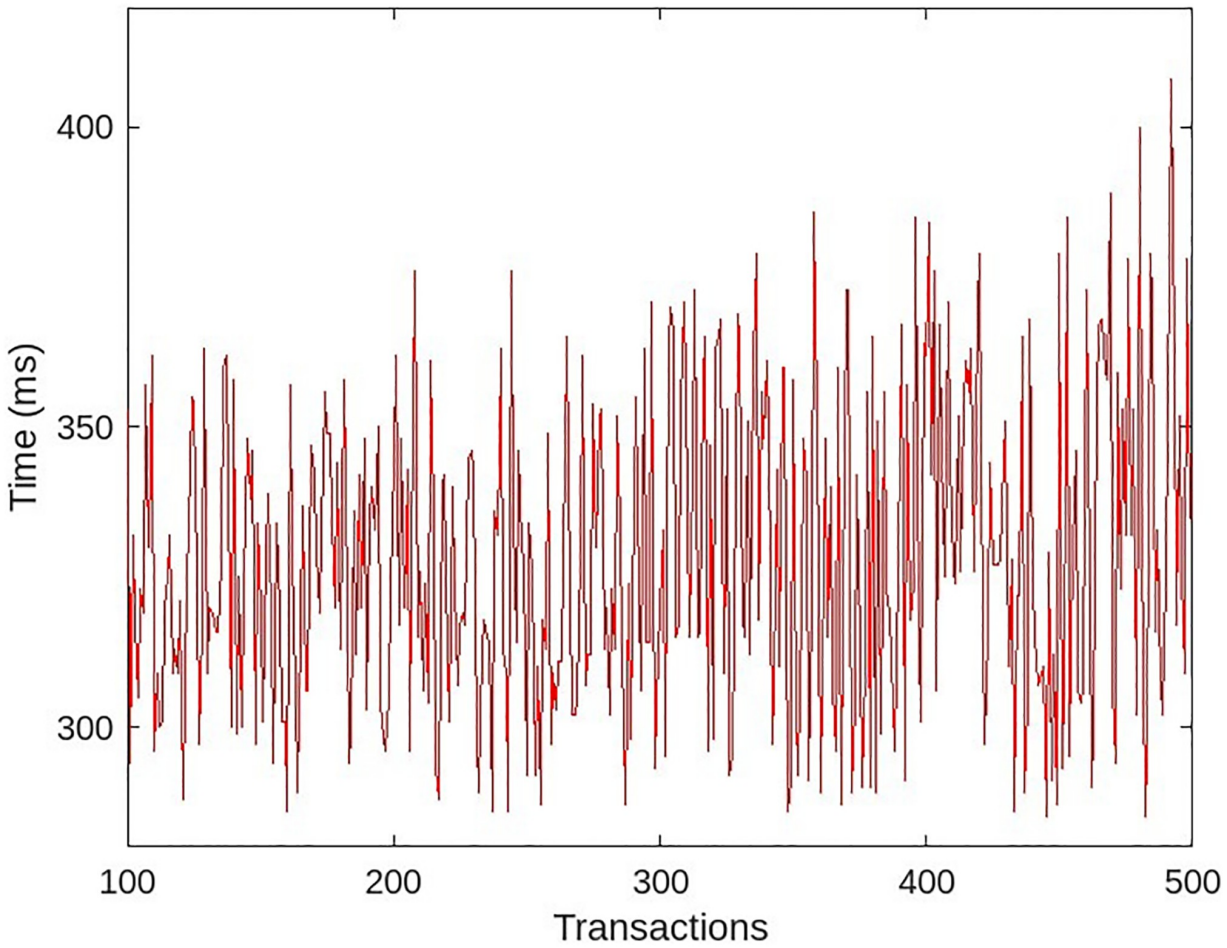
Accessing patient data with role-based access module.

A second experiment was conducted with the same data and user count. This time, we included an implementation module for role-based access rights. All 30 users in the system had access to the patient medical data, and we randomly revoked the access rights of the medical users so that, at any given time, a maximum of 5 users had no access. Fig 12 depicts the latency of data access from the blockchain when a system demonstrates access rights capabilities.

**Fig 13:**
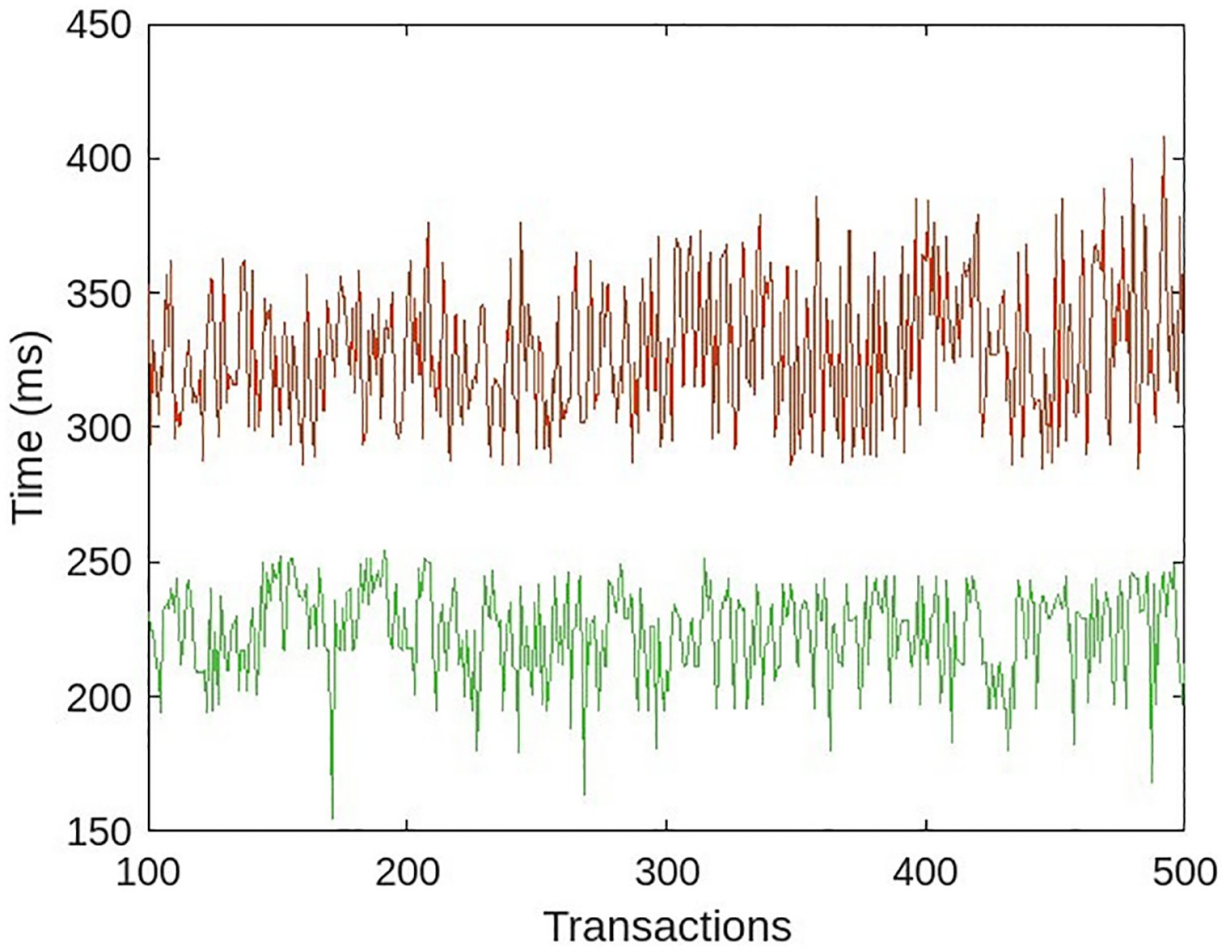
Comparative results of role-based access module.

When the system lacked an access rights module, all medical users accessed data at roughly the same amount of time, resulting in a uniform transaction time, as depicted by the green line in Fig 13. The slight variation in time is a result of the random network delay. Each transaction required additional processing time to compute the data bindings of the requesting medical user when the system had access rights. The red line in Fig 13 indicates that transactions required more time to process than in the absence of an access rights module.

The fluctuation in the red line is due to the fact that a small number of users have no access rights and the transaction is rolled back early with an error, whereas for users who have access to the data, the transaction was completed with the results, as well as a small random network delay. In both cases, as the number of transactions increases, the delay begins to increase. In order to achieve multi-entity authentication, each transaction is signed with the MHS’s trust certificate keys. As the queue begins to grow at MHS, where each request waits to be signed using trust certificate keys, the process becomes slower. In a medium-sized MHS, the number of requests for access to patient data is low; consequently, the queue is likely smaller, and the latency is negligible.

#### Effect of number of trusted entities in MHS

**Fig 14:**
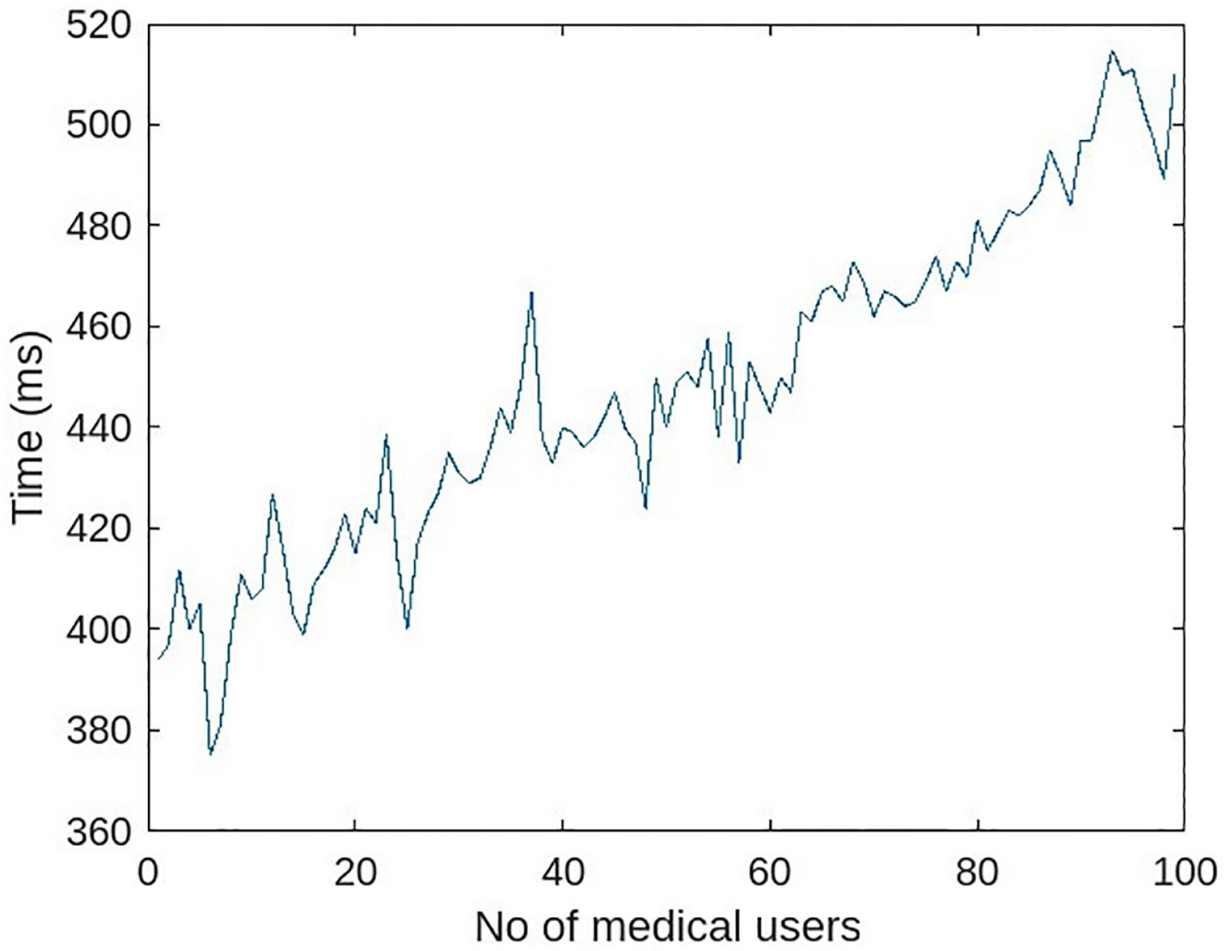
Response time with a varying number of trusted entities.

A series of experiments were conducted to determine the effect of the number of MHS medical users. The role-based access rights module was enabled, and 500 requests were sent per experiment in a unit of time. 40% of the requests were to read the patient’s medical history, while 60% of the requests were to read the patient’s current medical condition. There were an equal number of physicians, nurses, and assistants in each experiment. For the first experiment, 15 medical users were created in the MHS. In subsequent experiments, the number of medical users gradually increased to 99. The 10% of requests that violate access rights are sent for each experiment, 50 samples are taken for each experiment, and the average of each experiment is considered. The outcomes are depicted in Fig 14.

Results indicate that performance degrades as the number of users increases. This is a result of the increased number of bindings and keys used to sign requests. The selection of the appropriate binding from the list of available bindings necessitates searching, and trust keys are increased. The greater the number of transactions queued at MHS, waiting to be signed and sent on the blockchain. It takes time for the smart contract to identify the appropriate binding for the incoming request. The response time increased by 24 percent, or 60 milliseconds, with 400 requests per unit time for a medium-sized MHS with 30 medical users compared to a system without role-based access. Acceptable response time for a medium-sized MHS with 30 medical users. The proposed system achieves role-based authentication for multiple entities at the expense of minimal performance. The feature comparison of the proposed framework with benchmark systems is shown in Table 3.

**Table 3:** Comparison of functionality features with other blockchain-based authentication schemes.

Feature	[34]	[40]	[36]	[45]	[38]	Proposed
Dynamic rights	×	×	×	×	×	✔
Multi-entity authentication	×	×	×	×	×	✔
Single access revocation	×	✔	×	✔	×	✔
Multi-access revocation	×	×	×	×	×	✔
Role-based authentication	×	×	×	✔	×	✔
DoS attack	N/A	✔	N/A	N/A	N/A	✔
Reply attack	N/A	✔	✔	N/A	N/A	✔
Man-in-the-middle attack	N/A	✔	✔	N/A	N/A	✔
Mutual authentication	✔	✔	✔	×	×	✔
Confidentiality	✔	✔	✔	✔	✔	✔
Privacy	✔	✔	✔	✔	✔	✔

## Conclusion

Biosensors share data across different data users in healthcare institutions which raises security concerns. This study proposed and successfully implemented blockchain-based multi-entity authentication and role-based authorization for accessing the owner’s medical data. The system ensures authentication, data privacy, non-repudiation, and the ease with which access to data can be granted or revoked. The outcome demonstrates that the proposed model exhibits security in the context of the three metrics addressed in this study with minimal processing cost and a 24% delay. The proposed authentication and access control scheme along with other base techniques does not detect the malicious activities of users who are authorized, machine learning techniques such as CNN can be applied to detect such activities to provide more secure access control. In a dynamic environment, the owner of medical data can easily connect his medical devices to the healthcare system. The proposed framework ensures that data remain confidential and that the data owner retains exclusive control over his medical data at all times. In a highly dynamic work environment, the system becomes a bit costly. As smart contacts and role bindings took place on the blockchain, new blocks were created every time access was granted or revoked. This frequent addition of blocks in the blockchain network raises the running costs i.e. gas fee which is paid by the data owner. Also when the number of medical users and data features on which the data owner wants to set access rights increase, the processing delay increases which was noticed as non-linear. A better binding method may be proposed in the future which can solve the problem of non-linearity in processing.
